# αA Crystallin May Protect against Geographic Atrophy—Meta-Analysis of Cataract vs. Cataract Surgery for Geographic Atrophy and Experimental Studies

**DOI:** 10.1371/journal.pone.0043173

**Published:** 2012-08-20

**Authors:** Peng Zhou, Hong-Fei Ye, Yong-Xiang Jiang, Jin Yang, Xiang-Jia Zhu, Xing-Huai Sun, Yi Luo, Guo-Rui Dou, Yu-Sheng Wang, Yi Lu

**Affiliations:** 1 Department of Ophthalmology, Eye and ENT Hospital of Fudan University, Shanghai, People's Republic of China; 2 Department of Ophthalmology, Xijing Hospital, Fourth Military Medical University, Xi'an, People's Republic of China; Massachusetts Eye & Ear Infirmary, Harvard Medical School, United States of America

## Abstract

**Background:**

Cataract and geographic atrophy (GA, also called advanced “dry” age-related macular degeneration) are the two major causes of visual impairment in the developed world. The association between cataract surgery and the development of GA was controversial in previous studies.

**Methods/Principal Findings:**

We performed a meta-analysis by pooling the current evidence in literature and found that cataract is associated with an increased risk of geographic atrophy with a summary odds ratio (OR) of 3.75 (95% CI: 95% CI: 1.84–7.62). However, cataract surgery is not associated with the risk of geographic atrophy (polled OR = 3.23, 95% CI: 0.63–16.47). Further experiments were performed to analyze how the αA-crystallin, the major component of the lens, influences the development of GA in a mouse model. We found that theαA-crystallin mRNA and protein expression increased after oxidative stress induced by NaIO_3_ in immunohistochemistry of retinal section and western blot of posterior eyecups. Both functional and histopathological evidence confirmed that GA is more severe in αA-crystallin knockout mice compared to wild-type mice.

**Conclusions:**

Therefore, αA-crystallin may protect against geographic atrophy. This study provides a better understanding of the relationship between cataract, cataract surgery, and GA.

## Introduction

Cataract and age-related macular degeneration (AMD) are the two major causes of visual impairment in the developed world [1]. Cataract is a clouding that develops in the crystalline lens of the eye. Cataract surgery is currently one of the most frequently performed and successful surgical procedures [2]. Advanced AMD has two major subtypes: geographic atrophy (GA, also called advanced “dry” AMD) and choroidal neovascularization (also called “wet” AMD) [3]. Geographic atrophy [4]–[6] is characterized by confluent areas of cell death in photoreceptors and retinal pigment epithelium, is bilateral in more than half of patients, and is responsible for 10% of the cases of legal blindness resulting from age-related macular degeneration. Both cataract and GA are strongly age related.

The association between cataract surgery and the development of GA was controversial in previous studies. In the Beaver Dam Eye Study (BDES) [7], a positive cross-sectional association was found between cataract surgery and GA. The association was consistent with findings in the Los Angeles Latino Eye Study (LALES) [8], but not with findings in the Blue Mountains Eye Study (BMES) [9] or the Age-related Eye Disease Study (AREDS) [10]. However, the positive association between cataracts and GA was consistent in the Beaver Dam Eye Study [7] and LALES [8].

The pathogenesis of the association between cataract surgery and GA is less clear. The previous hypothesis is that cataract removal results in increased risk because the cataract, a barrier to ultraviolet radiation, has been removed [11]. Based on this hypothesis, in theory, the prevalence of GA should be decreased in patients with cataract. However, the truth is that the prevalence of GA is higher in patients with cataract than the control. Therefore, this hypothesis is not sufficient to explain the clinical phenomenon.

One common change involving cataracts and cataract surgery has been neglected: the change of α-crystallins, which are the major protein of lens. The α-crystallins are small heat shock proteins which play central roles in maintaining lens transparency and refractive properties [12]. The discovery in 1992 that these proteins possess chaperone-like activity has led most researchers to focus on the ability of α-crystallins to prevent protein aggregation in vitro. While the ability of α-crystallins to efficiently trap aggregation-prone denatured proteins in vitro is thought to delay the development of age-related cataracts in vivo, α-crystallins have additional functions which may also contribute to cataract pathology. In addition to chaperone activity, α-crystallins are known to protect cells from stress-induced apoptosis, regulate cell growth, and enhance genomic stability [13]. They also physically and functionally interact with both the cell membrane and cytoskeleton. Functional changes in α-crystallin have been shown to modify membrane and cell-cell interactions and lead to lens cell pathology in vivo [14].

Because most studies on geographic atrophy and cataract surgery or cataracts had relatively small sample sizes, we combined pieces of evidence from the published literature for a meta-analysis. In this study, we preformed a meta-analysis focusing on the association between GA and cataracts or cataract surgery. Furthermore, the function of α-crystallins was studied in a GA animal model to investigate its role in the development of GA.

## Materials and Methods

### Meta-analysis

This meta-analysis followed the PISMA statement guidelines. [15] The meta-analysis of the association between GA and cataract or cataract surgery was performed as we have described previously [16]. In brief, relevant studies were selected by searching PubMed and Web of Science database (updated to December 5, 2011) using the following search terms: (“cataract” OR “cataract surgery”) AND (“age-related macular degeneration” OR “geographic atrophy”) with the limit “humans”. All the resulting studies were retrieved, and their references were checked for other relevant publications. Review articles were also searched to find additional eligible studies. Only studies published in English with full-text articles were included in this meta-analysis. We did not define any minimum number of patients for a study to be included in the meta-analysis. For overlapping studies, only the one with the latest published was included.The identified articles were read carefully and assessed independently by two of the authors (Zhou, P and Lu, Y), and any discrepancies in their eligibility were adjudicated by Sun, XH.

The inclusion criteria were: (1) evaluating the association between the cataract and risk of geographic atrophy; (2) evaluating the association between the cataract surgery and risk of geographic atrophy; and (3) with sufficient available data to estimate an OR with its 95% CI. The included studies had to meet [(1)+(3)] or [(2)+(3)] of the above-mentioned criteria. Any study with internally inconsistent data was excluded. The following variables were extracted from each study, if available: first author's surname, publication year, numbers of cases and controls, and numbers of cases and controls in different groups whenever possible. Information was carefully and independently extracted from all the eligible publications by two of the authors (Zhou, P and Lu, Y). Disagreement was resolved by discussion between the authors. If they could not reach a consensus, another investigator (Sun, XH) adjudicated over the disagreements. (Figure S1, Table S1)

ORs and their 95% CIs were obtained directly or calculated from the data given in the articles. A random- or a fixed-effects model was used to calculate the pooled effect estimates in the presence (P<0.10) or absence (P>0.10) of heterogeneity, respectively. The potential publication bias was examined visually in a funnel plot of log [OR] against its standard error (SE), and the degree of asymmetry was tested using Egger's test (P<0.05 considered to be statistically significant). We conducted influence analysis by omitting each study to find potential outliers. All of the statistical analyses were performed using Stata/SE version 11.0 (Stata Corporation, College Station, TX).

### Animals

The 129S6/SvEvTac wild-type mice were purchased from Chinese Scientific Academy (Shanghai, China), while the αA-crystallin knockout mice in 129S6/SvEvTac background [17] were obtained from the National Eye Institute. Mice between 6 and 8 weeks old were fed the standard laboratory chow in an air-conditioned room equipped with a 12-hour light/12-hour dark cycle. All procedures were performed in compliance with the Fudan University Institutional Animal Care and Use Committee's approved protocols and the ARVO Statement for the Use of Animals in Ophthalmic and Vision Research.

### Chemical formulation and experimental procedure

Mice were briefly restrained. Sodium iodate (NaIO_3_; Sigma, St. Louis, MO) was diluted with Phosphate buffered saline (PBS) solution and 20 mg/kg body weight of NaIO_3_ was injected through the tail vein. [18] Animals injected with equivalent volumes of PBS solution served as controls. Electroretinography and fundus photographs were assessed at 21 days post injection. After the tests were made, mice were euthanized with CO_2_ and their eyes processed for histology.

### Experimental groups

The mice were divided into four groups: control wild type (PBS-treated WT), NaIO_3_-treated wild type (NaIO_3_-treated WT), control αA-crystallin knockout mice (PBS-treated αA−/−), and NaIO_3_-treated αA-crystallin knockout mice (NaIO_3_-treated αA−/−). Each group consisted of seven mice in the age range of 6–8 weeks.

### Electroretinography (ERG)

Mice were dark-adapted overnight and anesthetized by intraperitoneal injection of ketamine (100 mg/kg body weight) and xylazine (10 mg/kg body weight). Pupils were dilated with topical administration of 2.5% phenylephrine containing 0.5% tropicamide, and the cornea was anesthetized with 0.5% proparacaine. Scotopic ERGs were measured from dark-adapted mice using a low-intensity stimulus, and mesopic ERGs were measured using a non-attenuated light stimulus. To measure cone responses, a 6 lux white background light was delivered through the other arm of the coaxial cable to suppress rod responses, and a non-attenuated light stimulus was applied. The a-wave amplitude was measured from the baseline to the trough of the a-wave, while b-wave amplitude was measured from the trough of the a-wave to the peak of the b-wave.

### Histopathologic analysis

For histopathologic analysis, eyes were enucleated, and the anterior poles were removed. The remaining eyecups were snap-frozen in tissue-freezing medium (Triangle Biomedical Sciences, Durham, NC). Sections (8 µm) were stained with hematoxylin and eosin (H&E) to assess the histopathologic changes of retina.

### Western blot analysis

The western blot was performed as described previously. [19], [20] Lysed posterior eyecups, including retina and RPE, were centrifuged at 11,000 g for 20 minutes. Supernatants were collected, and proteins were resolved on Tris-HCl 10% polyacrylamide gels (Ready Gel; Bio-Rad, Hercules, CA) at 120 V. The proteins were transferred to PVDF blotting membrane (Millipore, Bedford, MA). The membranes were probed with antibody for αA-crystallin (Stressgen, San Diego, CA) and GAPDH (Millipore) at 1∶1,000 dilution. Membranes were washed and incubated with a horseradish peroxidase (HRP)-conjugated secondary antibody (1∶3,000, Vector Laboratories, Burlingame, CA) for 30 min at room temperature. Images were developed by adding ECL chemiluminescence detection solution (Amersham Pharmacia Biotech, Cleveland, OH).

### Immunohistochemistry

Immunohistochemical stains were performed on freezing tissue sections. After blocking with 20% normal donkey serum for 30 minutes, slides were probed overnight at 4°C with primary αA-crystallin antibody (Stressgen, San Diego, CA) diluted into blocking solution. Slides were incubated with biotinylated goat anti-rabbit immunoglobulin Ig G (1∶500) for 1 h at room temperature. The signal was detected using streptavidin-conjugated horseradish peroxidase and peroxidase activity was visualized with diaminobenzidine/H2O2 (Vector Laboratories, Burlingame, CA). After color development, slides were counterstained with hematoxylin.

### Reverse transcription PCR and relative quantitative real-time PCR

The reverse transcription PCR (RT-PCRwas performed as described previously. [16] Total RNA was isolated using a TRIzol reagent (Invitrogen, Carlsbad, CA, USA). The first strand of cDNA was synthesized with 1 µg of total RNA, oligo(dT) primer and AMV reverse transcriptase (Promega, Madison, WI, USA). The primers used in RT-PCR were as follows: CRYAA forward: 5′- TTTTGAGTATGACCTGCTGCC - 3′, reverse: 5′- TGGAACTCACGGGAAATGTAG -3′; GAPDH, forward: 5′- GAAGGTGAAGGTCGGAGTC -3′, reverse: 5′- GAAGATGGTGATGGGATTTC -3′. [21] The specificity of the PCR amplification products was checked by performing dissociation melting-curve analysis and by 1% agarose gel electrophoresis. Quantification analysis of CRYAA mRNA was normalized with a housekeeping gene, GAPDH, as an internal control.

### Isolation of primary cultured RPE cells from mice

Primary cultured RPE cells were isolated from knockout mice as previously described. [22] The cornea, lens, vitreous, and retina were removed from eyes soaked in phosphate buffered saline (PBS) containing 5% penicillin/streptomycin (Sigma). The choroid/sclera tissue was then placed into a 2% dispase solution in PBS for 20 min at 37°C. After the incubation, the tissue was rapidly pipetted up and down for 30 s. The dispase solution containing the RPE cells was passed through a 70 µ filter followed by a 40 µ filter after which the cells were spun down and resuspended in Ham's F-12 Media (Cellgro, Herndon, VA) containing at least 25% fetal bovine serum (FBS; Irvine Scientific, Santa Ana, CA). The RPE were then grown on laminin coated plates (Becton Dickinson and Company, Franklin Lakes, NJ).

### Detection of ROS Production

To determine the compartmentalized accumulation of reactive oxygen species (ROS), cells on an eight-well Lab-TekTM chamber were stained with carboxy-H2-DCFDA (Molecular Probes; 5 µM for 1 h at 37°C), and rapidly evaluated by confocal microscopy (LSM510, Zeiss, Thornwood, NY, USA). A green color is observed when ROS accumulated. Nucleus were stained with propidium Iodide (PI) as red fluorescence.

### Statistics

All experiments were repeated at least three times. Values in the figures were expressed as means ± SE. Statistical analyses were performed with Student's *t* test. Values in the figures are expressed as means±SE. Values of P<0.05 were considered statistically significant.

## Results

### Meta-analysis shows that cataract, not cataract surgery, is associated with an increased risk of geographic atrophy

We performed a meta-analysis focusing on the relationship between geographic atrophy and cataract or cataract surgery. There were four published articles [7]–[10] investigating the association between cataract surgery and geographic atrophy risk (Figure S1). Two studies investigated the association between cataract and geographic atrophy risk [7], [8] (Figure S1). Table 1 lists the main characteristics of relevant studies.

**Table 1:** Characteristics of studies included in meta-analysis.

No.	Year	First Author	Studies	Cataract surgery				Cataract			
				surgery		control		cataract		control	
				(n = 988)		(n = 21,918)		(n = 1,969)		(n = 12,924)	
				GA	No GA	GA	No GA	GA	No GA	GA	No GA
1	2002	Klein	BDES	3	94	17	3328	8	562	11	2718
2	2006	Cugati	BMES	4	128	51	2271	N.A.	N.A.	N.A.	N.A.
3	2008	Chew	AREDS	14	525	205	5383	N.A.	N.A.	N.A.	N.A.
4	2010	Fraser-Bell	LALES	4	216	9	10654	5	1394	8	9287

BDES = Beaver Dam Eye Study;

LALES = Los Angeles Latino Eye Study;

BMES = Blue Mountains Eye Study;

AREDS = Age-related Eye Disease Study;

N.A. = not available.

**Figure 1:**
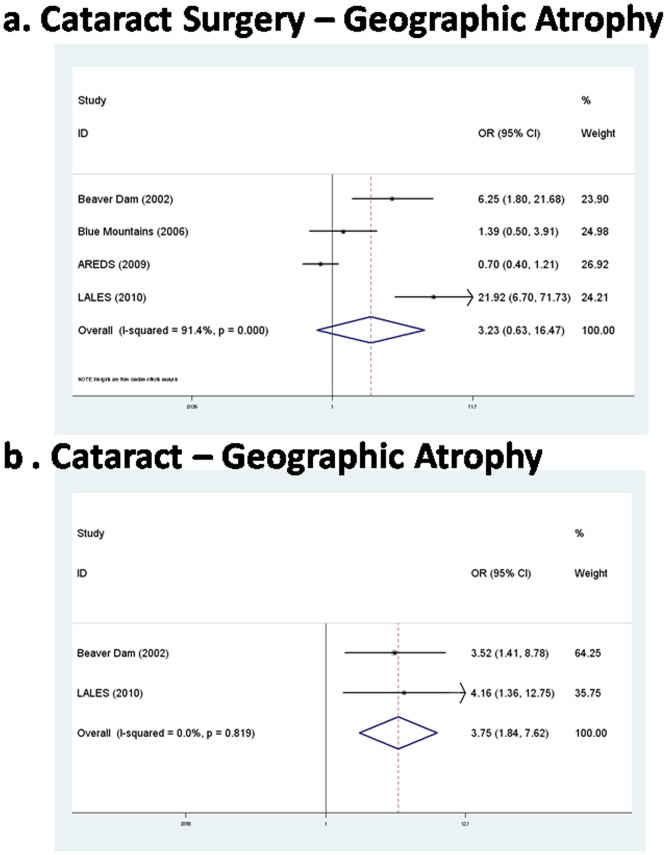
The forest plot of meta-analysis. Each study is shown by the point estimate of the odds ratio (OR) (the size of the square is proportional to the weight of each study) and the 95% confidence interval (CI) for the OR (extending lines). a. the association of cataract surgery with geographic atrophy; b. the association of cataract with geographic atrophy. LALES = Los Angeles Latino Eye Study; BMES = Blue Mountains Eye Study; AREDS = Age-related Eye Disease Study.

Positive association between cataract and risk of geographic atrophy was found (polled OR = 3.75, 95% CI: 1.84–7.62, *P* = <0.001). However, no significant association between cataract surgery and risk of geographic atrophy was found (polled OR = 3.23, 95% CI: 0.63–16.47, *P* = 0.159). (Figure 1, Table 2).

**Table 2:** Pooled ORs of TLR3 1234C>T in different genetic models.

	*Ph*	Polling Model	Polled OR (95%CI)	*P*
**Cataract surgery - GA**	<0.001	Random M-H	3.23 (0.63–16.47)	0.159
**Cataract - GA**	0.819	Fixed M-H	3.75 (1.84–7.62)	<0.001

*Ph = P* value of *Q* test for heterogeneity test;

OR = odds ratio;

CI = confidence interval.

M-H = Mantel-Haenszel.

### Reduced ERG amplitudes in NaIO_3_-treated αA-crystallin knockout mice

**Figure 2:**
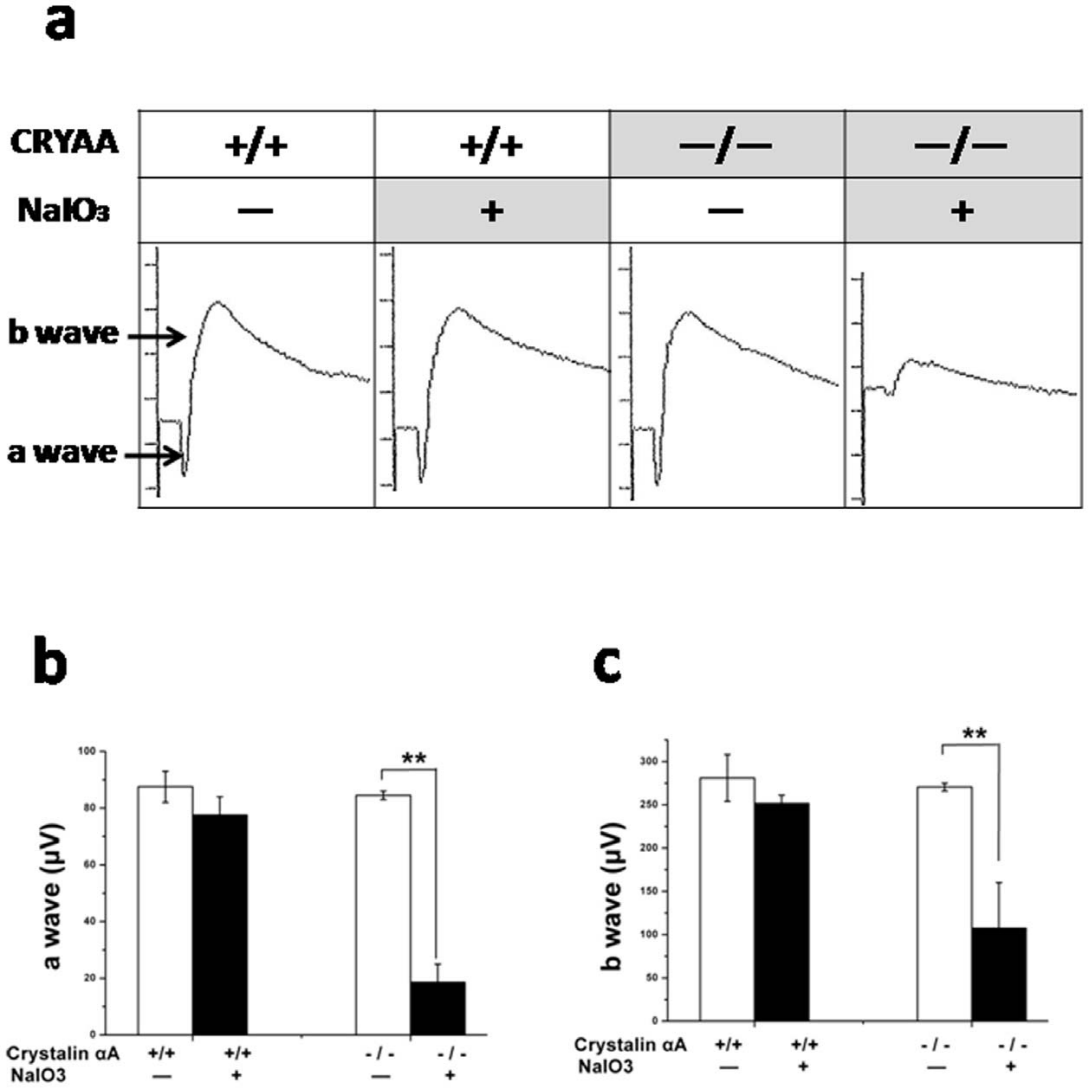
Reduced ERG amplitudes in NaIO_3_-treated αA-crystallin knockout mice. The amplitudes of a-wave of the ERG of NaIO_3_-treated αA−/− mice decreased to 21.89% of that of PBS-treated αA−/− mice. The amplitudes of b-wave of the ERG of NaIO_3_-treated αA−/− mice decreased by 60.26%, compared with that of PBS-treated αA−/− mice. Data are mean ± SEM, n = 5/group, **P<0.01.

To determine whether the absence of αA-crystallin had an effect on the retinal function of NaIO_3_-induced retinal degeneration, we compared mesopic (mixed rod and cone) ERG responses of the four groups of mice (PBS-treated WT, NaIO_3_-treated WT, PBS-treated αA−/−, and NaIO_3_-treated αA−/−) at the end of 3 weeks. Significant differences were observed in the ERGs of NaIO_3_-treated αA−/− mice compared with the PBS-treated αA−/− mice (Figure 2). No significant differences were found between the ERGs of the NaIO_3_-treated and control in wild-type mice.

The amplitudes of a-wave of the ERG of NaIO_3_-treated αA−/− mice decreased by 78.11% of that of PBS-treated αA−/− mice. The amplitudes of b-wave of the ERG of NaIO_3_-treated αA−/− mice decreased by 60.26%, compared with that of PBS-treated αA−/− mice. Generally speaking, a-wave of ERG demonstrates the function of the outer layer of the retina, while b-wave shows that of the inner layer. The ERG results suggested that the function of the outer layer of retina was more suppressed than that of the inner layer.

### Histopathological evidence for accelerated NaIO_3_-induced degeneration in αA-crystallin knockout mice

**Figure 3:**
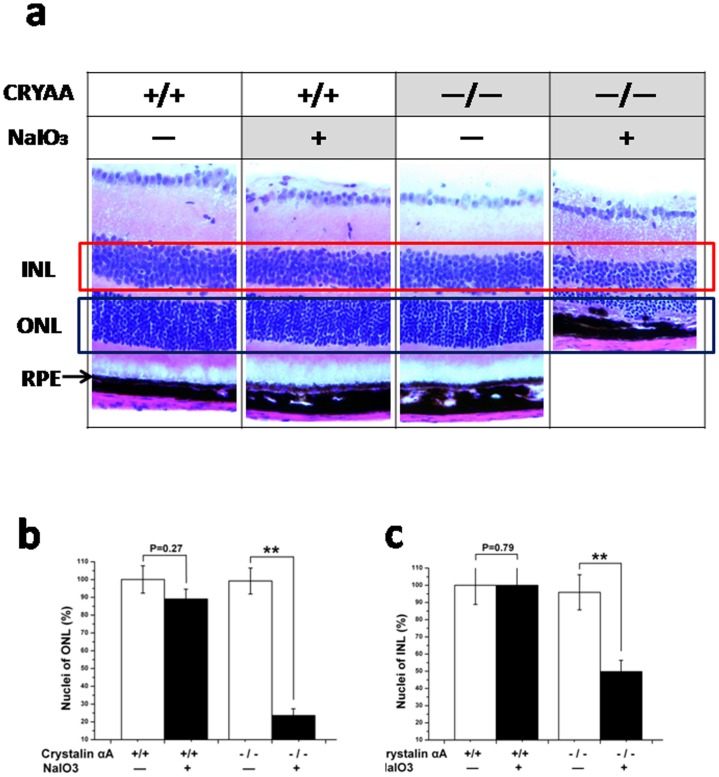
Histopathological evidence for accelerated NaIO_3_-induced degeneration in αA-crystallin knockout mice. Retinas from αA-crystallin knockout mice revealed more severe degeneration from NaIO_3_ injection as compared to wild-type retinas. The loss of nuclei was more prominent in αA-crystallin knockout retina vs. that of wild type. The decrease in the number of nuclei per unit area was statistically significant with NaIO_3_ injection only in the outer nuclear layer of the crystallin knockout mice. No significant differences of nuclei numbers were found between the NaIO_3_-injected and PBS-injected in wild-type mice. The RPE layers were discontinuous and damaged in αA-crystallin knockout mice with NaIO_3_ injection, while they were continuous in wild-type mice. CRYAA = αA-crystallin; INL = inner nuclear layer; ONL = outer nuclear layer; RPE = retinal pigmental epithelium; **P<0.01.

Retinas from αA-crystallin knockout mice revealed more severe degeneration from NaIO_3_ injection as compared to wild-type retinas. An assessment of the extent of retinal damage by NaIO_3_ was made by counting the number of nuclei in the inner nuclear layer, outer nuclear layer, and ganglion cell layer of wild type and αA-crystallin knockout retinas. This semi-quantitative analysis revealed that the loss of nuclei was more prominent at 3 weeks post injection of NaIO_3_ in αA-crystallin knockout retina vs. that of wild type. The decrease in the number of nuclei per unit area was statistically significant (p<0.01) with NaIO_3_ injection only in the outer nuclear layer of the crystallin knockout mice. No significant differences of nuclei numbers were found between the NaIO_3_-injected and PBS-injected in wild-type mice (Figure 3).

The RPE layers were discontinuous and damaged in αA-crystallin knockout mice with NaIO_3_ injection, while they were continuous in wild type mice.

### Increased αA-crystallin expression after NaIO_3_ treatment

**Figure 4:**
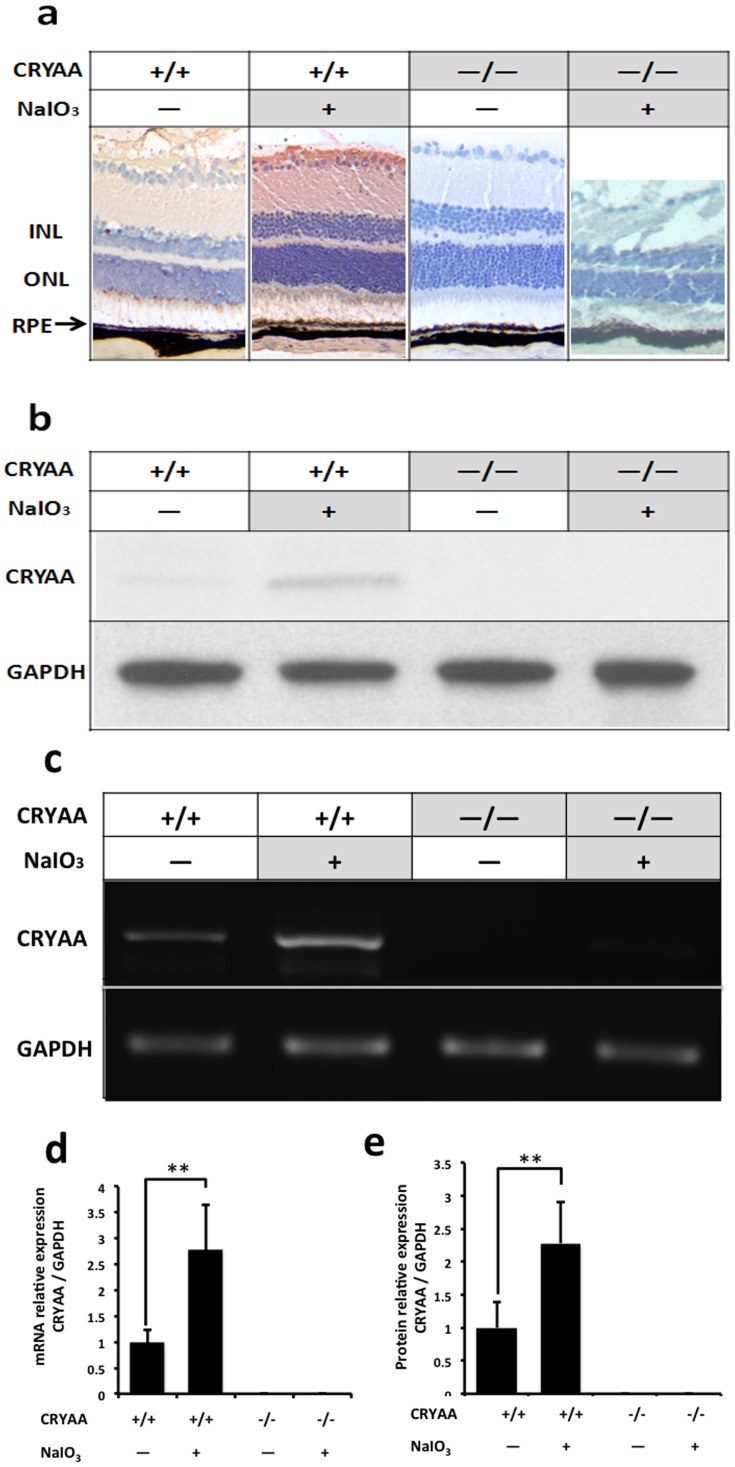
Increased αA-crystallin expression after NaIO_3_ treatment. Immunohistochemistry staining showed that αA-crystallin in retina increased after NaIO_3_ treatment. Protein expression of αA-crystallin of mice eye cup after NaIO_3_ treatment was examined by Western blot analysis. αA-crystallin prtein increased to 2.28±0.62 times comparing to control (p<0.01), while mRNA increased 2.79±0.86 times comparing to control (p<0.01). No αA-crystallin was detected in αA-crystallin knockout mice. CRYAA = αA-crystallin, **P<0.01.

Immunohistochemistry staining showed that αA-crystallin in retina increased after NaIO_3_ treatment. (Figure 4a) Protein expression of αA-crystallin of mice eye cup after NaIO_3_ treatment was examined by Western blot analysis. αA-crystallin increased to 2.28±0.62 times comparing to control (p<0.01). No αA-crystallin protein was detected in αA-crystallin knockout mice. (Figure 4b, d) mRNA expression of αA-crystallin of mice eye cup after NaIO_3_ treatment was examined by RT-PCR analysis. αA-crystallin increased to 2.79±0.86 times comparing to control (p<0.01). No αA-crystallin mRNA was detected in αA-crystallin knockout mice. (Figure 4c, e) All experiments were repeated at least three times.

### Increased accumulation of ROS in CRYAA −/− RPE cells

**Figure 5:**
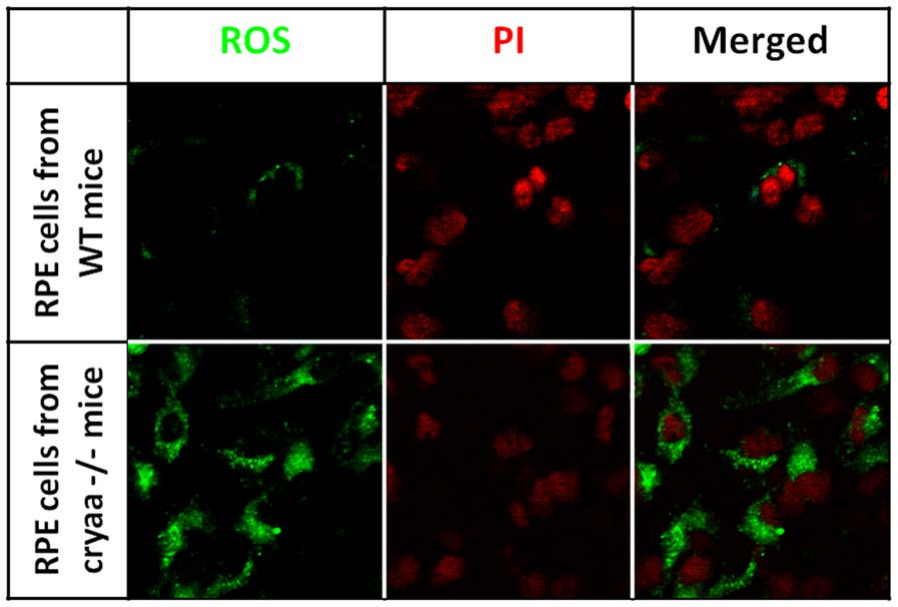
Increased accumulation of ROS in CRYAA −/− RPE cells. Primary cultured RPE cells was isolated from knockout mice and treated with NaIO3. Confocal images show ROS accumulation in RPE cells. The accumulation of ROS was much stronger in RPE cells from αA-crystallin knock our mice than that from wild type mice. These results show that knock out αA-crystallin results in increased accumulation of ROS in RPE cells treated with NaIO3.

Primary cultured RPE cells was isolated from knockout mice and treated with NaIO_3_. Figure 5 shows confocal images of ROS accumulation in RPE cells. The accumulation of ROS was much stronger in RPE cells from αA-crystallin knock our mice than that from wild type mice. These results show that knock out αA-crystallin results in increased accumulation of ROS in RPE cells treated with NaIO_3_.

## Discussion

The meta-analysis shows that cataract, not cataract surgery, is associated with an increased risk of geographic atrophy. Further experiments found that αA-crystallin may play an important role in this association.

Only a small number of publications could be included in the meta-analysis. The overall scientific level of evidence of these articles was not high. There were no trials at the highest level of evidence, which are randomized controlled trials (RCT). It needs to be mentioned that randomizing patients to not undergo cataract surgery when their vision is poor enough to affect their daily life would neither be ethical nor practicable. Thus, observational cohort studies and non-randomized clinical trials are probably the best possible evidence. Both are rated as a level 2 on the five-step hierarchy of levels of evidence for medical studies [23].

The results of our meta-analysis is not consistent with previous theory that increased risk or progression of GA (dry age-related macular degeneration) after cataract surgery is related to the increased exposure of the retina to short-wavelength light [11], [24], [25]. However, the meta-analysis of 22,906 patients did not find significant association between cataract surgery and GA. Instead, we found that cataract is associated with an increased risk of geographic atrophy. That means that the risk of GA increased in cataract patients, although they had cataract as a barrier to short-wavelength light. Therefore, the short-wavelength light damage theory is not sufficient to explain our meta-analysis findings.

αA-Crystallin, which is a member of the small heat shock protein (sHSP also known as the HSP20) family, plays an important neuroprotection function in retina. [22], [26]–[28] We analyzed cataracts and cataract surgery, and found that they have a common change: the crystallins in the lens. Cataracts cause crystallins to degenerate, and cataract surgery removes the lens, the largest bank of crystallins from the eyes. Furthermore, experiments were performed to analyze the effect of αA-crystallin on GA in a mouse model. We found that αA-crystallin expression increased after oxidative stress induced by NaIO3. Both functional and histopathological evidence confirmed that GA is more severe in αA-crystallin knockout mice compared with wild-type mice.

Dry AMD is a challenging disease to study because of its complex genetics, late onset, and confounding environmental risk factors. As with other human diseases, finding a model system for AMD has been a demanding task. The pathology of dry AMD consists of degeneration of photoreceptors and the RPE, lipofuscin accumulation, and drusen formation. Genetically engineered mice have been used for generating models that simulate human AMD features. However, most mice develop retinal lesions at an older age (6–24 months). [29] Furthermore, there is no CRYAA −/− genetically engineered dry AMD mice. Therefore, we tried to find another way to mimic the dry AMD. Oxidative stress plays an important role in the pathogenesis of dry AMD. [30] NaIO3 can induce oxidative stress. NaIO3 damages mostly the central pole of the retina in low dose. Such a pathological phenomenon resembles those observed in dry AMD in their clinical course that initially involve the central part of the retina and spread gradually. Therefore, chemical damage induced by NaIO3 mimics dry AMD in humans and may serve as a model useful for studying retinal damage. [31] Therefore, we selected this model in our study.

The association of cataract with an increased risk of geographic atrophy can be explained by our findings. The pathogenesis of cataract is the degeneration of crystallins [32]. αA-crystallin is the major protein of the lens [17]. Neal's study showed that the concentration of alpha-A crystallins in vitreous is decreased in cataract patients. [33] After having cataract, the αA-crystallin degenerates. The total of active αA-crystallin decreases. Therefore, those people with cataract are more susceptible to have GA compared with people without cataract in the same dose of oxidative stress.

The phenomenon that cataract surgery is not associated with an increased risk of geographic atrophy can also be explained by our findings. Neal et. al reported that the concentration of αA-crystallin in vitreous is increased after cataract surgery [33]. Increased αA-crystallin may protect retina from GA. The protective αA-crystallins is produced by “after cataract”. Over the year, many patients who have an uncomplicated routine cataract surgery will developed a clouding or a thickening of a natural membrane in the eye which sits just behind the lens implant, called the posterior lens capsule. This has been referred to as “after-cataract” or a secondary capsular opacification. The “after cataract” will produce new αA-crystallins and leak into vitreous.

In this study, ERG findings, the a-wave amplitude decreased more than that of b-wave in knockout mice exposed to oxidative stress. This suggests that the function of the outer retina was more suppressed than the inner, in relation to controls. However, only outer retinal atrophy was seen in histopathological findings. This phenomenon may because that the changes of function are earlier than that of anatomy. [34] ERG may be a better test to evaluate the retinal response to oxidative stress damage.

Some limitations of this meta-analysis should be acknowledged. First, this study involves two subjects, human patients with unknown genetic background and mice with clear genetic defects. However, there is not a sub-population of human subjects with congenital aA-Crystallin defects, which may share similarity to the aA-crystallin KO mice and contribute the correlations between lens and retina degenerating defects.

We put forward a hypothesis that the eye has an oxidation/anti-oxidation homeostasis. αA-crystallin is an important anti-oxidation factor. In young people, the αA-crystallin is sufficient to fend off oxidative stress damage, so their eyes do not have GA. When people have cataracts, the degenerative αA-crystallin cannot protect against oxidative stress damage, and they get GA. After cataract surgery, the concentration of αA-crystallin increases in the vitreous, and those eyes have more anti-oxidation factors. They are less likely to have GA than those who did not have cataract surgeries.

## Supporting Information

Figure S1 **Flow diagram of papers accepted and rejected during selection procedure.** Five - hundred-seventy-seven potentially relevant studies were identified from the PubMed and Web of Science database of which 6 were eligible for our review.
(DOC)

Table S1 **Checklist of items to include when reporting a systematic review or meta-analysis.**
(DOC)
