# Supplementary material for: αA Crystallin May Protect against Geographic Atrophy—Meta-Analysis of Cataract vs. Cataract Surgery for Geographic Atrophy and Experimental Studies
Source: PLoS One. 2012 Aug 20;7(8):e43173. doi: 10.1371/journal.pone.0043173 (PMC3423426; doi:10.1371/journal.pone.0043173)
Supplement: Figure S1 — Flow diagram of papers accepted and rejected during selection procedure. Five - hundred-seventy-seven potentially relevant studies were identified from the PubMed and Web of Science database of which 6 were eligible for our review. (DOC) [file pone.0043173.s001.doc]

**Identification**

**Screening**

**Eligibility**

**Included**

Records identified through database searching

(n=556)

Additional records identified through other sources

(n=21)

Records screened on abstract

Records excluded (n=516)

Full-text articles assessed for eligibility

(n=26)

Excluded (n=22)

Only wet AMD, n=16

GA data did not include, n=8

Studies included in quantitative synthesis (meta-analysis):

**Cataract Surgery vs. GA**

**(n=4)**

Records after duplicates removed (n=542)

Studies included in quantitative synthesis (meta-analysis):

**Cataract vs. GA**

**(n=2)**

**Figure S1- Flow diagram of papers accepted and rejected during selection procedure**
